# Manipulation of Growth and Architectural Characteristics in Trees for Increased Woody Biomass Production

**DOI:** 10.3389/fpls.2018.01505

**Published:** 2018-10-16

**Authors:** Victor B. Busov

**Affiliations:** School of Forest Resources and Environmental Science, Michigan Technological University, Houghton, MI, United States

**Keywords:** hormones, transcription factors, woody biomass growth, molecular mechanisms, crown architecture, adventitious rooting, tree biotechnology

## Abstract

Growth and architectural traits in trees are economically and environmentally important and thus of considerable importance to the improvement of forest and fruit trees. These traits are complex and result from the operation of a number of molecular mechanisms. This review will focus on the regulation of crown architecture, secondary woody growth and adventitious rooting. These traits and processes have significant impact on deployment, management, and productivity of tree crops. The majority of the described work comes from experiments in model plants, poplar, apple, peach, and plum because these species allow functional analysis of the involved genes and have significant genomics resources. However, these studies convincingly show conserved mechanisms for elaboration of specific growth and architectural traits. The conservation of these mechanisms suggest that they can be used as a blueprint for the improvement of these traits and processes in phylogenetically diverse tree crops. We will specifically consider the involvement of flowering time, transcription factors and hormone-associated genes. The review will also discuss the impact of recent technological advances as well as the challenges to the dissection of these traits in trees.

## Introduction

Intensive forest plantation can alleviate the harvesting pressure on native forests via allowing production of the same or larger amount of wood on a much smaller land base (Paquette and Messier, 2010). Improved genetics through breeding is one, if not the leading factor in this increased productivity (Fenning and Gershenzon, 2002; Ruotsalainen, 2014). However, tree breeding is slow due to long generation times, traits that need a long time to evaluate and complex genetic architecture of these traits (Fenning and Gershenzon, 2002). Understanding the involved genetic mechanism could significantly accelerate the process through both conventional breeding and genetic engineering.

Here we review the current knowledge about the molecular mechanisms that underpin three developmental processes in trees with significant impact on intensive plantation deployment, management and growth. The review will focus on mechanisms and genes that can provide positive effects and thus are of breeding value rather than exhaustively discuss progress in the dissection of each process. Where available, the reader will be pointed to reviews that deal with these processes in a more comprehensive manner.

## Manipulation of Crown Architecture

Crown architecture is a compound trait resulting from the position, size, periodicity, angle and density of the branches. Crown characteristics affect plantation density, interception of photosynthetic light and quality of the derived wood. Depending on the plantation purpose, the direction and extent to which these characteristics need to be changed can vary. Branches originate in axillary meristems (AMs) and thus establishment and outgrowth of AMs has a profound effect on branch characteristics and crown architecture. AM initiation is exclusively characterized in herbaceous plants and there is no information about the effect of these genes in trees. We therefore will not cover here these developments. Excellent reviews on AM initiation discuss in detail these genes and mechanisms (Janssen et al., 2014; Yang and Jiao, 2016).

### Branch Outgrowth

**FIGURE 1:**
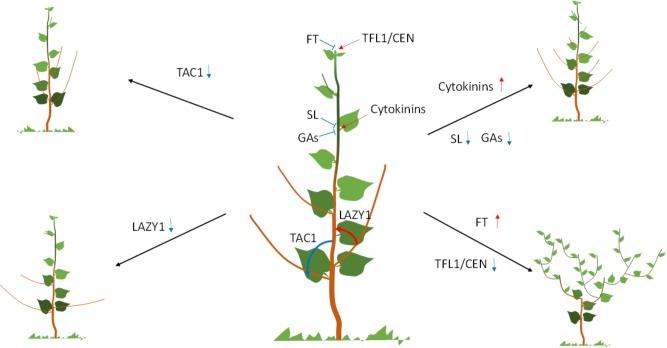
Regulation of crown architecture in trees. Diagram in the middle shows a WT tree with the different regulators and their putative roles indicated. The tree drawings to the left and right shows the effect of the different regulators on the crown architecture. Arrows show positive while blunted line negative effect. Vertical up red arrow indicates upregulation, while blue down arrow downregulation of the gene.

Once established, the AM outgrowth is typically suppressed, a phenomenon known as apical dominance. Auxin is central to the establishment and maintenance of apical dominance (**Figure 1**). The regulatory roles of auxin in apical dominance are indirect and are explained by the canalization and secondary messengers’ models (Domagalska and Leyser, 2011; Teichmann and Muhr, 2015). However, only the latter provides genes and mechanisms manipulated in trees and is thus covered here. According to this model, auxin synthesized in the shoot apex, moves basipetally to the roots to generate a secondary signal that travels acropetally to regulate bud outgrowth. Cytokinin was the first candidate for a second messenger because it has a strong positive effect on axillary bud outgrowth when exogenously applied (**Figure 1**). However, cytokinin acropetal transport was not able to activate bud outgrowth (Faiss et al., 1997). This led to the discovery of the shoot branching hormone strigolactones (SLs). SLs have strong negative effects on bud outgrowth (**Figure 1**), are synthesized in roots, acropetally transported to shoots and biosynthetic genes are positively regulated by auxin. SLs metabolic and signaling genes are strong regulators of bud outgrowth in a number of plant species including several trees (Domagalska and Leyser, 2011; Muhr et al., 2016; Foster et al., 2018). RNAi knockdown of poplar and apple orthologs of SLs biosynthetic genes resulted in increased sylleptic branching (branches developed from lateral buds that have not undergone dormancy) (Muhr et al., 2016; Foster et al., 2018).

### Branch Angle

Significant progress has been made in trees in elucidating the mechanism underpinning branch angle characteristics. Using an innovative sequencing approach and a distinct peach mutant with acute branch angle, the causative gene was isolated to be *TILLER ANGLE CONTROL1* (*TAC1*) (Dardick et al., 2013). TAC1 was originally found to control tiller angle in rice (Yu et al., 2007). *TAC1* belongs to a small family of genes. All the genes in the family, characterized to date in several plant species, including trees (poplar and plum) control branch or lateral root angles (**Figure 1**; Xu et al., 2017; Hollender et al., 2018). Depending on presence of a conserved domain, members of the family can increase (*TAC1*) or decrease (*LAZY1*) branch angles (**Figure 1**; Dardick et al., 2013; Hollender and Dardick, 2015; Xu et al., 2017).

### Roles of Gibberellins

Gibberellins control stem elongation, but can also regulate crown characteristics (**Figure 1**). *Gibberellin 2-oxidase* (*GA2ox*) overexpression leads to low levels of bioactive GAs, and proliferation of long sylleptic branches at a wide, almost perpendicular angle to the main stem (Mauriat et al., 2011; Zawaski et al., 2011). After 2 years in the field, *GA2ox* overexpressors produced a wide oval crown (Zawaski et al., 2011). A similar effect was also observed in turf grass and rice (Agharkar et al., 2007; Lo et al., 2008). These effects are possibly mediated via the GAs regulation of PIN auxin efflux carrier abundance (Willige et al., 2011; Lofke et al., 2013; Mauriat et al., 2014). In contrast, modifications of GA signaling via DELLA domain proteins produces a highly compact crown consisting of short branches with narrow acute angle (Zawaski et al., 2011). The effect of DELLA domain proteins on branching may be due their interactions with the transcription factor BRANCHED1 (Daviere et al., 2014).

### Flowering and Crown Architecture

The determinacy of the meristem is genetically programmed, heritable and significantly affects plant architecture, including crown characteristics in trees (McGarry and Ayre, 2012). Indeterminate meristems typically produce monopodial growth characterized by a pronounced primary stem. In contrast, plants with determinate meristems show sympodial growth, a process of repeated loss of the shoot apical meristem (SAM) through terminal differentiation and lateral outgrowth from the axillary meristem resulting in a compound shoot architecture. Monopodial and sympodial growth types result from differences in the expression of genes and localization of proteins from the CENTRORDIALIS/TERMINAL FLOWER/SELF PRUNING (CETS) family that control flowering (McGarry and Ayre, 2012). CETS genes form a small gene family in Arabidopsis and other plant species. Very small (few amino acid) changes in the sequence of the proteins can reverse their function (Hanzawa et al., 2005). For example, FT promotes, while a close family member, TFL1 inhibits flowering (Hanano and Goto, 2011). FT is a mobile signal originating in the leaf that moves through the phloem stream to reach the shoot or axillary meristems and initiates terminal flower development (Pin and Nilsson, 2012). TFL1 plays an antagonistic role to FT in the SAM (Kobayashi et al., 1999). Low and high FT/TFL1 ratio in the SAM results in indeterminate and determinate growth respectively, (McGarry and Ayre, 2012; **Figure 1**). This model has been confirmed through transgenic overexpression of *FT* orthologs in several tree species (Hsu et al., 2006, 2011; Srinivasan et al., 2012; Klocko et al., 2016). FT overexpression leads to early flowering and highly branched, sympodial growth. Increase in FT/TFL1 balance via downregulation of TFL1/CEN genes in apple leads to similar effects as with *FT* overexpression (Kotoda et al., 2006; Flachowsky et al., 2012). RNAi downregulation of two *TFL1/CEN*-like homologs in poplar (*PopCEN1* and *PoCEN2*) produced similar but more moderate flowering and architectural phenotypes (Mohamed et al., 2010). Interacting factors and regulators of FT and TFL1/CEN, can also produce changes in tree architecture. Overexpression of CsRAV1, a chestnut ortholog of *TEMPRANILLO*, a regulator of FT (Castillejo and Pelaz, 2008), led to upregulation of a poplar ortholog of *FT* (*PttFT2*) expression (Triozzi et al., 2018) and consequently to increased branching both under greenhouse and field conditions (Moreno-Cortes et al., 2012, 2017). Under field conditions, increased branching led to increased biomass (Moreno-Cortes et al., 2017). Similarly, overexpression of a poplar ortholog of GIGANTEA, a positive regulator of *FT*, upregulated *PttFT2* and increased sylleptic branching in poplar (Ding et al., 2018). FT interacts with FD to promote flowering and overexpression of poplar *FD* homolog led to precocious flowering and sympodial, highly branched growth (Parmentier-Line and Coleman, 2015).

## Increase of Secondary Woody Growth

**FIGURE 2:**
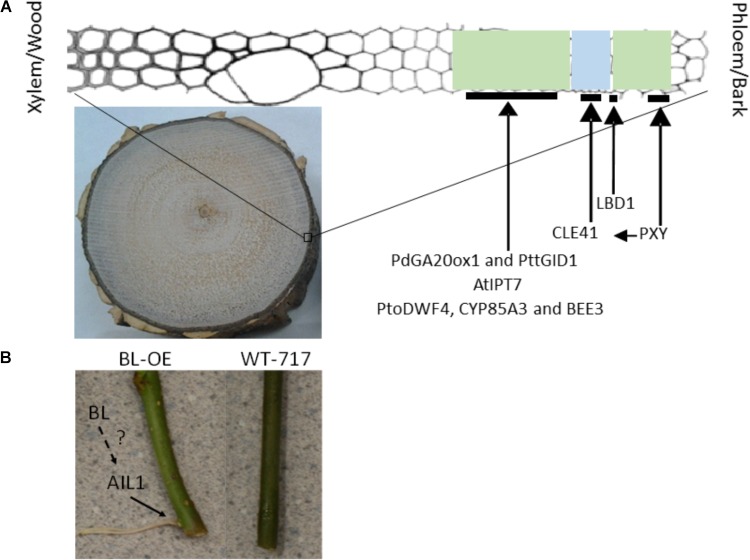
Genes increasing secondary woody growth **(A)** and adventitious rooting **(B)** in trees. **(A)** Picture on the bottom shows a cross section from a from a 3-year-old poplar tree, approximately 10 cm in diameter, showing an extensive wood production in the middle and bark at the periphery. The diagram on the top shows the cambium zone with the developing xylem and phloem. Green highlighted area indicates developing xylem and phloem. Blue highlight indicates cambium zone. The black lines show the approximate presumed location where the positive regulators of secondary growth play critical roles. **(B)** Picture to the left is a transgenic plant overexpressing BL (see text for detail) and to the right WT-717. See Yordanov et al., 2017 and the text for further details. The dotted line shows the presence of additional unknown regulators, indicated by a question mark.

Secondary growth originates in a lateral meristem known as vascular cambium, which in trees shows exaggerated and perennial activity, compared to herbaceous plants, resulting in production of massive amounts of conductive and supportive tissues, referred to as wood (Helariutta and Bhalerao, 2003; Elo et al., 2009; Barra-Jimenez and Ragni, 2017). Bifacial periclinal division of the cambium cells, followed by growth and differentiation results in production of phloem/bark to the outside and xylem/wood to the inside of the tree trunk (Helariutta and Bhalerao, 2003; Zhang et al., 2014). Excellent reviews comprehensively discuss the process (Groover, 2005; Demura and Fukuda, 2007; Groover et al., 2010; Spicer and Groover, 2010; Mizrachi and Myburg, 2016). Here we focus on genes and mechanisms that have positive effects on secondary woody growth and thus are of potential breeding/improvement value (**Figure 2A**). The only exception would be the genes that affect bark development, Bark is typically considered as waist byproduct and thus decrease of bark production would be favored. However, notable exceptions where bark increase would be the goal would be special plantation for production of cork as well as breeding for resistance to pests, fires and drought.

### Gibberellins

The first demonstration of increased secondary growth was via transgenic modifications of gibberellin biosynthesis (Eriksson et al., 2000). Overexpression of the Arabidopsis GA-20 oxidase (GA20ox), a key biosynthetic enzyme, resulted in significant (2-fold) increase in wood production (**Figure 2**). Similarly, overexpression of pine *PdGA20ox1* in poplar resulted in nearly 3-fold increase in woody biomass (Jeon et al., 2016; **Figure 2A**). In addition, overexpression of the poplar orthologs of the GA receptor *PttGIBBERELLIN-INSENSITIVE DWARF1* (*PttGID1*) resulted in similar wood biomass enhancement (Mauriat and Moritz, 2009; **Figure 2A**). Increase in bioactive gibberellins also increased fiber length and cellulose/xylan content (Eriksson et al., 2000; Jeon et al., 2016). Increase in GA signaling, however, did not result in changes in fiber length. The increased GA synthesis and signaling in the transgenics poplars, had negative effect on root development, decreased expression of defense-related genes and resulted in poor leaf development (Eriksson et al., 2000; Mauriat et al., 2014; Jeon et al., 2016). These negative pleiotropic effects resulting from the constitutive overexpression were mitigated by using a xylem-specific promoter (Jeon et al., 2016). The xylem specific expression resulted in similar increases of wood biomass (Jeon et al., 2016). However, tissue-specific upregulation of *PttGID1* using a different xylem-specific promoter did not result in any increases in woody biomass (Mauriat and Moritz, 2009), suggesting that different promoter::gene combination can have specific effects. Cisgenic modifications of several poplar GA 20-oxidase genes led to no pleiotropic effects and increased wood biomass and fiber length but the gains were more modest than these obtained with the constitutive and xylem-specific promoters (Han et al., 2011).

### Cytokinins

The regulatory role(s) of cytokinins during secondary woody growth has been known (Nieminen et al., 2008). However, it was only recently demonstrated that modification of cytokinin biosynthesis can have a positive effect on secondary woody growth (Immanen et al., 2016). Transgenic poplars transformed with the Arabidopsis *AtIPT7* (key cytokinin biosynthetic gene) driven by a xylem-specific promoter, showed significant (nearly 2-fold) increases in secondary growth and no negative pleiotropic effects (Immanen et al., 2016) (**Figure 2A**).

### Brassinosteroids

Recent evidence suggests that both brassinosteroid (BR) biosynthesis and signaling has a positive effect on woody biomass production (Noh et al., 2015; Jin et al., 2017; Shen et al., 2018; **Figure 2**). Overexpression of key biosynthetic genes (*PtoDWF4* and *CYP85A3*) led to increased brassinosteroid concentrations and woody biomass (Jin et al., 2017; Shen et al., 2018). The productivity gains however, were much smaller than, these observed with the manipulations of GA and cytokinin biosynthesis/signaling. The increase in brassinosteroids led to longer fibers and no or little impact on cell wall chemistry (Jin et al., 2017; Shen et al., 2018). Similar results were obtained with manipulation of brassinosteroid signaling. Overexpression of a poplar ortholog of *BEE3* (*Brassinosteroid Enhanced Expression 3*), a transcription factor involved in BR signaling increased stem, leaf and root biomass. As with the enhancement of BR biosynthesis, gains in wood biomass were less than these observed with GA and cytokinin and ranged between 25 and 50%. The modifications of both BRs biosynthesis and signaling did not cause any negative pleiotropic effects, despite the strong constitutive promoters used in both studies.

### Small Protein Signaling in the Cambium

Cambial cell division in Arabidopsis is controlled by a protein ligand receptor complex (Fisher and Turner, 2007; Hirakawa et al., 2008; Etchells and Turner, 2010). The ligand is the small CLE41 protein, produced in the phloem and transported into the cambium, where it interacts with the PXY receptor to stimulate cambium cell division (Fisher and Turner, 2007; Hirakawa et al., 2008; Etchells and Turner, 2010). Recently, constitutive expression of aspen orthologs of the ligand and receptor in transgenic poplar trees resulted in highly pleiotropic and negative effects on growth and tissue organization (Etchells et al., 2015). However, when the ligand and receptor were simultaneously upregulated in their native tissue domains employing tissue specific promoters, not only that the negative effects were completely mitigated, but also the double transgenic plants showed a nearly double increases in wood production (**Figure 2A**).

### Secondary Phloem and Bark Development

Secondary growth also yields secondary phloem and bark. Using activation tagging in poplar, the first gene that regulates secondary phloem development was discovered (Yordanov et al., 2010). The gene encodes a transcription factor of the LATERAL ORGAN BOUNDARIES (LBD) gene family that is a positive regulator of secondary phloem development (Yordanov et al., 2010; Yordanov and Busov, 2011). Transgenic plants overexpressing the gene produced more, while dominant negative modification of the protein produced less secondary phloem (**Figure 2A**).

## Genes Promoting Adventitious Rooting

Adventitious rooting (AR) is root formation from organs and tissues that typically do not produce roots. The process is most important in forestry and horticulture for clonal propagation and deployment of elite germplasm. The cellular and molecular events underlying AR has been reviewed elsewhere (Diaz-Sala, 2014; Legue et al., 2014; Pacurar et al., 2014). Here we focus on several genes that have been functionally characterized in trees and provide strong positive effects on AR formation.

### Controls of Cell Proliferation Provide Points for AR Manipulation

AINTEGUMENTA (ANT) and ANT-like (AIL) genes are a group of eight AP2 transcription factors in Arabidopsis with important functions in regulation of meristem establishment and maintenance as well as organ growth and size (Horstman et al., 2014). One of the members of the *AIL* family from poplar (*AIL1*), showed induction during AR primordia activation (Rigal et al., 2012) and overexpression of the gene caused increase (**Figure 2B**), while RNAi downregulation decrease in the number of ARs. AIL1 transcriptionally regulates *Cyclin D3.1* by binding to its promoter (Karlberg et al., 2011). Thus, AIL1 promotes AR at least in part via activation of cell proliferation.

The BIG LEAF/STERILE APETALA (BL/SAP) gene from poplar has a positive effect on AR formation when ectopically expressed (Yordanov et al., 2017; **Figure 2B**). BL is an F box protein that regulates leaf size in poplar and Arabidopsis through control of cell proliferation (Wang et al., 2016; Li et al., 2018). BL/SAP targets proteins for degradation that negatively regulate AIL genes (PLETHORA 1 and 2) (Horstman et al., 2014; Wang et al., 2016; Li et al., 2018). Thus, BL likely regulates AR formation through promoting degradation of a repressor(s)of the AIL-like genes (**Figure 2B**), which has a positive effect on cell proliferation and meristem organization.

Both *AIL1* and *BL*, when overexpressed have significant pleiotropic effects (Rigal et al., 2012; Yordanov et al., 2017) and to serve as biotechnological tool for increased AR formation, will require inducible or tissue-specific upregulation.

### Gibberellins

GAs inhibit AR likely thought interfering with polar auxin transport (Mauriat et al., 2014). Increase and decrease in GA biosynthesis and signaling leads to decreased and increased AR (Busov et al., 2006; Gou et al., 2010; Elias et al., 2012). As mentioned earlier, GAs have strong positive effects on secondary woody growth and thus the decrease of AR may present an impediment for the clonal propagation of transgenics with increased GAs biosynthesis. Alternatively, decrease in bioactive GAs and block of signaling, which promotes AR formation, leads to various levels of dwarfism. Dwarfism is a desirable trait in fruit and ornamental tree crops and the increased AR formation would provide an additional benefit for the propagation of these genotypes. In forestry, however, extreme dwarfism may lead to loss in biomass productivity and thus, this effect can be either mitigated via increased girth growth using gene stacking with other transgenes that promote radial expansion (see above) or selection of semi-dwarfism genotypes (Elias et al., 2012).

### Activation Tagging Discovery of AR-Involved Genes

Using activation tagging (AT), the poplar gene ETHYLENE RESPONSE FACTOR 3 (ERF003) was shown to have positive effect on AR formation (Trupiano et al., 2013). In addition to ERF003, several other AT mutants affected in AR and associated with ethylene signaling and biosynthesis were also discovered (Trupiano et al., 2013). These genes however, have not been recapitulated through re-transformation experiments and thus their involvement and utility in manipulation of AR formation is still tentative.

## Future Outlook

### Improvements in Transformation Technologies

Transformation is the golden standard for asserting gene function and preferred method of choice in delivering advanced editing tools like CRIPSR/Cas9 system (Busov et al., 2005; Altpeter et al., 2016; Ran et al., 2017). However, transformation technologies are slow, inefficient, require cumbersome tissue culture processes and remain largely genotype-specific (Busov et al., 2005; Altpeter et al., 2016; Baltes et al., 2017), even in genera considered as ‘easy-to-transform’ like poplars. Thus, major strides in understanding and improving transformation technologies are needed (Altpeter et al., 2016).

### Understanding Promoter Architecture

The need for research in isolation and engineering artificial promoters for precise targeting of transgenic manipulations has been known and well-recognized. However, research in this area has been lagging behind. The advances in gene editing and synthetic technologies would further necessitate better understanding promoter architecture in order to being able to effectively modify and design level and specificity of promoter activities.

### Application of CRISPR Technology

New technological advances in gene editing technologies like CRISPR/Cas9 promise to revolutionize tree improvement (Tsai and Xue, 2015). The CRISPR/Cas9 was successfully implemented in a poplar tree (Fan et al., 2015; Zhou et al., 2015). CRISPR/Cas9 compared to RNAi produced stronger and more uniform phenotypic effects when the same gene was targeted (Zhou et al., 2015). Although now, the majority of the CRISPR/Cas9 applications involve generation of knock-outs via non-homologous end joining, a significant progress is also made in the application of CRISPR/Cas9 for gene editing through homologous recombination (Schaeffer and Nakata, 2015). However, the latter is still in developmental stages for plants. CRIPSR/Cas9 can also alleviate the regulatory burdens associated with field-testing because, in some countries CRISPR/Cas9-modified genotypes are considered as a non-GMO type of modification.

### Using Induced and Natural Mutants

Application of natural or induced mutants in tree research has been rare. However, significant strides have been made in both approaches (Busov et al., 2010; Dardick et al., 2013). As described above, using activation tagging in poplar, genes important for secondary growth (Yordanov et al., 2010, 2014, 2017) and AR formation (Trupiano et al., 2013) were discovered. In addition to induced mutants, many natural tree mutants exist. The new sequencing technologies allow efficient mapping of the causative mutations, (Dardick et al., 2013). These approaches can be further used for identifying genes affecting various aspects of tree growth and development.

### Understanding Integrative System Controls

A significant progress has been made in identification of individual genes and pathways regulating different traits. However, it has been long known at an organismal level that the various processes are highly coordinated at tissue and organismal level but also in response to various environmental cues. Identification of the coordinating genes, signals and mechanisms can lead to more integrative manipulation of one or several traits.

## Author Contributions

The author confirms being the sole contributor of this work and approved it for publication.

## Conflict of Interest Statement

The author declares that the research was conducted in the absence of any commercial or financial relationships that could be construed as a potential conflict of interest.
